# Severe Lupus Enteritis in a Regional Hospital: An Uncommon Presentation of the Acute Surgical Abdomen

**DOI:** 10.1155/cris/1934052

**Published:** 2025-12-26

**Authors:** Emily Hammond, Samsher Ali

**Affiliations:** ^1^ Flinders and Upper North Local Health Network (FUNLHN), Whyalla, South Australia, Australia; ^2^ Central Adelaide Local Health Network (CALHN), Adelaide, South Australia, Australia

**Keywords:** damage control laparotomy, diagnostic imaging, early transfer, immunosuppression, severe dehydration, severe diffuse lupus enteritis

## Abstract

Systemic lupus erythematosus (SLE) is a multi‐system autoimmune disorder, with gastrointestinal (GI) involvement in 20%–50% of cases. Mostly, symptoms are non‐specific, but lupus enteritis is a rare acute manifestation occurring in less than 6% of patients with SLE. This infrequency often leads to a delayed diagnosis, resulting in significant morbidity and mortality. We present the case of a 40‐year‐old female with a 9‐year history of SLE, who developed severe diffuse lupus enteritis and hypovolemic shock in a regional hospital located 400 km from a tertiary care centre. The patient exhibited abdominal distension, vomiting and acute kidney injury. A contrast‐enhanced CT scan revealed circumferential bowel wall thickening and free fluid, consistent with lupus enteritis. Initial management focused on stabilisation through aggressive rehydration and monitoring while awaiting transfer to a tertiary facility. Corticosteroids and supportive care led to a gradual resolution of symptoms. This case is educational for rural surgeons, highlighting the importance of recognising and managing rare acute GI manifestations of collagen vascular diseases like SLE in resource‐limited settings. Early diagnosis and transfer are crucial to reducing mortality, and this case demonstrates the need for high clinical suspicion and decisive damage control intervention if indicated.

**Summary**

1. Early Recognition: Lupus enteritis should be suspected in Systemic lupus erythematosus (SLE) patients with acute abdominal symptoms, especially in rural settings where other causes of abdominal pain must be rapidly excluded.
2. Management in Resource‐Limited Settings: Stabilisation with aggressive rehydration and monitoring is essential in rural hospitals. Early transfer to tertiary centres is crucial to reduce morbidity and mortality.
3. Imaging findings: A contrast‐enhanced CT scan is crucial in the diagnosis of SLE enteritis. Look for the target sign (bowel wall oedema in concentric rings) and the comb sign (engorged mesenteric vessels), along with ascites. These findings, in the appropriate clinical context, strongly support lupus mesenteric vasculitis.
4. Surgical Preparedness: While surgery was avoided in this case, rural surgeons must be prepared for damage control laparotomy in selected cases where mesenteric ischaemic or bowel perforation is suspected. In such scenarios, urgent surgical intervention may be life‐saving, despite the high risks associated with operating in active SLE.

## 1. Introduction

Systemic lupus erythematosus (SLE) is a chronic multi‐system autoimmune disorder in which gastrointestinal (GI) involvement is relatively common, occurring in 20%–50% of cases. Most GI manifestations result in non‐specific symptoms, while a smaller number have significant symptoms and signs, which are suggestive of serious involvement. Notable GI manifestations of SLE include mesenteric vasculitis, protein‐losing enteropathy, intestinal pseudo‐obstruction, acute pancreatitis and autoimmune hepatitis [1]. Lupus enteritis, a rare but potentially life‐threatening manifestation of SLE, occurs in only 0.2%–5.85% of patients. Due to its rarity and overlap with other causes of acute abdominal pain, it is frequently overlooked in differential diagnoses, leading to delays in treatment and increased morbidity.

In rural and regional hospitals, where access to subspecialty care and advanced diagnostic tools is limited, early recognition of lupus enteritis is essential for reducing the risk of complications. This case report describes the unique challenges of managing a critically ill SLE patient with lupus enteritis in a regional hospital located 400 km from a tertiary care centre. By sharing this case, we aim to provide practical insights for rural surgeons and clinicians on the timely diagnosis, management and transfer of lupus enteritis and other complex autoimmune GI presentations. The need for a multidisciplinary approach, including diagnostic imaging and surgical preparedness, is critical in these scenarios.

## 2. Methods

We report the case of a 40‐year‐old female with a 9‐year history of SLE who presented to a regional hospital located 400 km from a tertiary care facility. Her medical history included lupus nephritis, synovitis, cutaneous involvement and serositis. She had been on a stable regimen of hydroxychloroquine and prednisolone, compliant until a few days before symptom onset.

### 2.1. Clinical Presentation

The patient had been unwell for 5 days before presenting to the hospital. She initially experienced mild diarrhoea that lasted ~24 h. Following this, she had not opened her bowels for 5 days but continued to pass flatus. Ongoing vomiting and an inability to tolerate oral intake were also reported. Her observations consisted of blood pressure of 110/70 mmHg, a pulse rate of 115 beats per minute and evidence of profound dehydration (decreased skin turgor and dry mucous membranes). The abdomen was distended and had central tenderness with no peritonism.

### 2.2. Laboratory Findings

Acute kidney injury (creatinine: 251 μmol/L), hypoalbuminemia (29 g/L), elevated LDH (406 U/L), mild anaemia (Hb: 118 g/L) and leukocytosis. The combination of high LDH and leukocytosis in this context raised concern for significant tissue stress or potential bowel ischaemia.

### 2.3. Imaging

**Figure 1:**
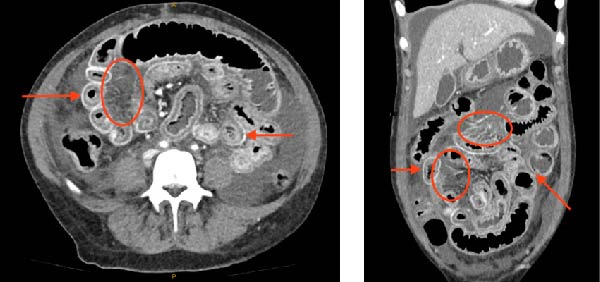
Portal phase CT abdomen of the patient acquired on presentation to the emergency department—as pictured, this demonstrated marked circumferential bowel wall thickening throughout the entire length of the small bowel and proximal colon (‘target sign’, demonstrated by red arrow). Engorgement of the mesenteric vessels (‘combs sign’) with attenuation of the mesenteric fat was also commented on (demonstrated by red circle), along with large volume free fluid. These signs are helpful in the identification of SLE enteritis [2].

A contrast‐enhanced CT revealed large volumes of abdominal free fluid, diffuse small bowel wall thickening along the entire length of the small bowel with abnormal enhancement—specifically with concentric bowel wall oedema producing the double‐halo appearance of ‘target sign’—and mild proximal dilation without occlusion or thrombosis. Engorgement of mesenteric vessels created the characteristic ‘comb sign’. These radiologic features are demonstrated in Figure 1 and are highly suggestive of SLE enteritis [3]. Adrenal hyper enhancement suggested a shocked state.

### 2.4. Management Approach

While it is important to accurately identify non‐SLE causes of acute abdominal pain, Janssens et al. [3] emphasise the need to maintain a high index of suspicion for SLE‐related causes, such as lupus enteritis, autoimmune pancreatitis and autoimmune hepatitis. The diagnostic challenge arises due to the significant overlap between SLE manifestations and non‐SLE conditions, making timely diagnosis difficult. In such cases, teleconsultation with subspecialists can be invaluable, providing additional insights and support in resource‐limited settings.

In this case, the need for surgery was excluded with telehealth input from a tertiary care centre, and the focus was maintained on rehydration and stabilisation while awaiting transfer to a tertiary care centre. However, in cases with alarm features—such as thrombocytopaenia, persistent leukocytosis or imaging findings, including pneumatosis intestinalis or free perforation—urgent surgical intervention may be required [4]. Thus, decision‐making should remain dynamic, with readiness for damage control laparotomy in deteriorating patients.

### 2.5. Final Diagnosis and Outcome

Upon transfer to the tertiary care centre, the diagnosis of diffuse lupus enteritis was confirmed by a multidisciplinary team, including rheumatologists. Upon arrival, she was treated with high‐dose intravenous corticosteroids (hydrocortisone equivalent to 100 mg QID), consistent with the standard of care for severe lupus enteritis [5]. This was gradually tapered over the following weeks. Symptomatic improvement was slow, requiring a 2‐week admission in a tertiary care service. Drainage of over 1 L of ascitic fluid provided symptomatic relief and confirmed exudative ascites with a serum ascites albumin gradient of 0.6 g/L. Regular abdominal X‐rays were utilised to monitor for radiological signs of deterioration or perforation. The colorectal team was consulted early and regularly for a surgical opinion to clinically exclude complications such as perforation and necrosis, as well as management of an associated ileus. Recovery was gradual; correction of electrolyte disturbances, bowel rest, corticosteroids and supportive ultimately stabilised both her enteritis and overall SLE activity. Despite a symptomatic recovery with resolution of her acute kidney injury and restoration of oral intake, her serum albumin levels remained below 22 g/L. Alpha‐1‐antitrypsin levels were sent to investigate for a concurrent protein‐losing enteropathy. In the interim, she was managed with a high‐protein diet and intravenous albumin replacement. After a 23‐day inpatient stay, she was discharged on oral corticosteroids and hydroxychloroquine.

The patient’s care was transitioned to outpatient rheumatology. Despite initial improvement, she re‐presented on two occasions within 2 months with recurrent abdominal symptoms and persistently low complement levels. She required further IV corticosteroid therapy and subsequently received rituximab as a steroid‐sparing agent. She ultimately underwent an endoscopy during her first re‐admission to exclude differentials. Endoscopic duodenal biopsies demonstrated non‐specific inflammation, reinforcing that histology is non‐specific in lupus enteritis. This pattern of relapse reflects the known recurrence risk of lupus enteritis, reported in up to a quarter of cases.

## 3. Research Ethics

This case report was exempt from ethical review by the SA Department for Health and Wellbeing Human Research Ethics Committee (DHW HREC), and written informed consent for publication was obtained from the patient.

## 4. Discussion

The accurate diagnosis and management of an acute abdomen in patients with SLE present significant challenges. This requires careful consideration of non‐SLE‐related pathologies, the side effects of SLE medications and GI manifestations of SLE itself, with one such manifestation, lupus enteritis, although rare, is life‐threatening and carries substantial risks of morbidity and mortality [6].

In an SLE patient presenting with an acute abdomen, it is critical to distinguish lupus enteritis from other more common causes of their symptoms. Principal considerations include mesenteric ischaemia, infectious gastroenteritis and mechanical small bowel obstruction. Mesenteric ischaemia more commonly afflicts individuals with cardiovascular risk factors, with compromise to mesenteric blood flow through an arterial thrombosis or embolus, leading to segmental infarction. In contrast, lupus enteritis stems from an immune‐mediated small‐vessel vasculitis, resulting in an absence of large‐vessel occlusion with a more diffuse pattern of bowel involvement [7]. Infectious enteritis may be bacterial, viral or parasitic in origin and usually features prominent diarrhoea, fever and a neutrophilic leukocytosis. By comparison, lupus enteritis often coincides with a systemic lupus flare and may be accompanied by other SLE manifestations such as serositis and nephritis [8]. Laboratory clues favouring SLE activity may include depressed complement levels or cytopaenias rather than an extreme neutrophil count [3]. Finally, a small bowel obstruction due to a mechanical cause, such as adhesions or a hernia, would typically show an abrupt transition point on imaging with proximal dilation and little mural enhancement. Lupus enteritis can mimic an obstruction through causing an inflammatory ileus—the radiological findings in our case demonstrated inflamed bowel but no discrete mechanical blockage. In practice, maintaining a broad differential and investigating for infection, vascular occlusion or surgical abdomen causes in parallel with SLE‐specific evaluations is the safest approach.

Differentiating between these various causes requires astute use of clinical assessments, laboratory investigations, and, most importantly, imaging studies. CT scans have proven particularly useful, often revealing characteristic findings (as noted above) that, when interpreted in the correct clinical context, are pivotal for diagnosis. A contrast‐enhanced CT may classically demonstrate a triad of diffuse bowel wall oedema with abnormal enhancement (‘target sign’), mesenteric vessel engorgement (‘combs sign’) and ascites. These radiological findings independently are present in over 70% of patients with lupus enteritis [3]. Radiological pneumatosis and biochemical abnormalities such as thrombocytopaenia, elevated LDH and rising creatinine should be recognised as potential markers of severity or impending surgical pathology, including intestinal necrosis or perforation [4]. Although rare, complications such as bowel necrosis, perforation, or protein‐losing enteropathy may occur, necessitating early escalation. Endoscopy has a limited diagnostic yield and should be reserved to rule out alternative aetiologies in cases of diagnostic uncertainty [3]. Endoscopic biopsies frequently fail to demonstrate definitive vasculitis or a specific inflammatory process, with the procedure carrying additional risk in actively inflamed bowel [9]. Thus, endoscopy is best reserved for excluding other aetiologies (such as infection or inflammatory bowel disease) when the diagnosis remains uncertain despite imaging and biochemical evaluation [10].

In rural and regional hospitals, where advanced imaging and specialist consultation are generally not immediately available, initial management should focus on stabilising the patient with rehydration, monitoring for signs of shock and ruling out the need for damage control surgery. Early involvement of tertiary centres—through telehealth or direct transfer—is critical. As this case illustrates, prompt multidisciplinary consultation and timely referral to a tertiary‐level centre can significantly reduce morbidity and mortality. Telemedicine consultations with sub‐specialists will invariably provide invaluable guidance in decision‐making and make the transfers efficient.

Equally important is planning for the long‐term management after the acute episode. Lupus enteritis has a propensity to relapse, with a documented recurrence rate of around 25% [3]. Rural healthcare providers should also be aware of the need for long‐term follow‐up, often involving a prolonged course of corticosteroids, to mitigate the risk of recurrence [11]. High‐dose corticosteroids will often require a prolonged taper and the addition of steroid‐sparing agents such as hydroxychloroquine, azathioprine, or rituximab to prevent future flares [5]. In our patient’s case, arrangements were made for close outpatient follow‐up under remote rheumatology supervision for medication titration, regular clinical assessments and monitoring of inflammatory markers and SLE serologies. This vigilant follow‐up strategy is essential in managing lupus enteritis, as early recognition of relapse and timely intensification of therapy can prevent serious complications. Despite these plans, the patient experienced recurrent symptoms requiring brief re‐admissions for intravenous corticosteroids and management of malnutrition; however, these admissions were brief.

This case reinforces the need for rural surgeons to be prepared for complex presentations, not just for lupus enteritis but for other similar autoimmune‐related conditions. While surgery was not required in this case, rural‐based surgeons must be familiar with and remain ready for damage control laparotomy in appropriately highly selected cases. It is worth noting that, in general, surgical resection for generalised systemic illnesses is associated with poor outcomes [12]. Therefore, the decision to operate must be balanced carefully against the risks, and if possible, the patient should be stabilised medically and transferred early. Further research into the damage control laparotomy for non‐traumatic abdominal emergencies, including lupus enteritis, is needed. Additionally, enhancing access to diagnostic tools and fostering strong networks for clinical/operative advice and early transfer to tertiary care centres will remain pivotal in improving the survival and quality of care for patients with rare, life‐threatening abdominal manifestations of autoimmune diseases.

## 5. Conclusion

Lupus enteritis, although rare, should be considered in any SLE patient presenting with an acute abdomen. In rural hospitals, early recognition, prompt stabilisation and timely transfer to tertiary care are crucial to prevent severe complications. Similar clinical and pathological processes are seen in other autoimmune and collagen vascular diseases patients with acute abdominal pain. In such scenarios, rural surgeons must maintain a high level of suspicion and readiness for surgical intervention. This case illustrates the vital role of rural healthcare professionals in managing complex cases and underscores the need for ongoing education, training and establishment of sustainable and long‐term networks with tertiary care centres in handling such rare conditions.

## Disclosure

The information provided in this case report has been carefully written and tailored by the authors to meet necessary standards and requirements.

## Conflicts of Interest

The authors declare no conflicts of interest.

## Funding

No funding was received for this work.

## Data Availability

Data sharing is not applicable to this article as no new data were created or analysed in this study.
